# Isolation and Molecular Typing of *Leishmania infantum* from *Phlebotomus perfiliewi* in a Re-Emerging Focus of Leishmaniasis, Northeastern Italy

**DOI:** 10.3390/microorganisms7120644

**Published:** 2019-12-03

**Authors:** Mattia Calzolari, Elena Carra, Gianluca Rugna, Paolo Bonilauri, Federica Bergamini, Romeo Bellini, Stefania Varani, Michele Dottori

**Affiliations:** 1 Istituto Zooprofilattico Sperimentale della Lombardia e dell’Emilia Romagna “B. Ubertini” (IZLER), Via Antonio Bianchi 9, 25124 Brescia, Italy; elena.carra@izsler.it (E.C.); gianluca.rugna@izsler.it (G.R.); paolo.bonilauri@izsler.it (P.B.); federica.bergamini@izsler.it (F.B.);; 2 Centro Agricoltura Ambiente “G. Nicoli”(CAA), Via Sant’Agata, 835, 40014 Crevalcore, Italy; rbellini@caa.it; 3 Department of Experimental, Diagnostic and Specialty Medicine, University of Bologna, via San Giacomo 14, 40126 Bologna, Italy; stefania.varani@unibo.it; 4 Unit of Microbiology, Regional Reference Center for Microbiological Emergencies (CRREM), St. Orsola Malpighi Hospital, University of Bologna, 40138 Bologna, Itally

**Keywords:** *Leishmania infantum*, *Phlebotomus perfiliewi*, *Phlebotomus perniciosus*, visceral leishmaniasis, molecular typing, parasite load, quantitative PCR

## Abstract

Visceral leishmaniasis (VL) caused by *Leishmania (L.) infantum* is a public health threat in the Emilia-Romagna region, northeastern Italy, but its epidemiology has not been fully elucidated in this area. The objective of this study was to characterize *Leishmania* infection in sand flies collected in a re-emerging focus of VL in the Bologna province. During the summer of 2016, 6114 sand flies were collected, identified, and tested for *Leishmania* detection. Of the identified sand flies, 96.5% were *Phlebotomus (P.) perfiliewi* and 3.5% were *P. perniciosus*. Detected parasites were characterized by biomolecular methods (multilocus microsatellite typing and characterization of repetitive region on chromosome 31), and quantified by real-time PCR. The prevalence of *Leishmania* infection in individually-tested *P. perfiliewi* sand flies varied from 6% to 10% with an increasing trend during the season. Promastigotes of *L. infantum* were isolated by dissection in one *P. perfiliewi* female; the isolated strain (Lein-pw) were closely related to *Leishmania* parasites from VL cases in northeastern Italy, but differed from strains isolated in dogs from the same area. Our findings strongly support the vector status of *P. perfiliewi* for human VL in the study area.

## 1. Introduction

In the Mediterranean basin, *Leishmania infantum* is the causative agent of visceral leishmaniasis (VL), a zoonotic parasitic disease affecting humans; dogs, susceptible to canine leishmaniasis, are considered the main reservoirs in this nosogeographical entity [1,2]. VL is endemic in Italy, where a peak of more than two hundred cases per year has been reported since the beginning of 2000 [3].

In Italy, the primary vector of VL is *Phlebotomus (P.) perniciosus*, a sand fly widely distributed across the country and particularly abundant in the Tyrrhenian and Southern regions [3]. Conversely, the sand fly, *P. perfiliewi*, more abundant in Central Italy [4], is considered a less relevant vector of *L. infantum* in restricted foci of leishmaniasis [5,6].

**Figure 1:**
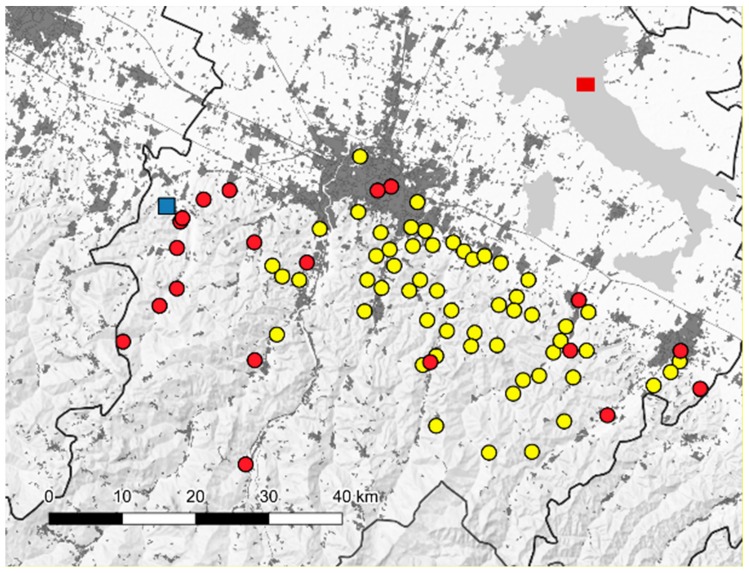
Map of monitored area in the Bologna province, with reference to the location on the Italian map. Blue square: isolation site of *Leishmania infantum* Lein-pw. Yellow circles: visceral leishmaniasis cases reported in 1971–1972 [7]. Red circles: visceral leishmaniasis cases reported in 2012–2013 [8]. In grey: urban areas.

After the VL epidemic in 1971–1972 with 60 cases [7], recrudescence of human leishmaniasis was recorded in 2012–13 in the hilly part of the Bologna province (Emilia-Romagna region, northeastern Italy), with more than 30 cases [8] (Figure 1). The sand fly, *P. perfiliewi,* is largely predominant in this area, reaching high density in the sylvatic environment in the central-eastern part of the region, while *P. perniciosus* is present at considerably lower density [9], this particular proportion between the two species is also confirmed by samplings dating back to the 70s [10,11].

Molecular methods are increasingly employed for epidemiological purposes to detect or to confirm *Leishmania* infection, to characterize the parasites at the species or population level in hosts and vectors, but also in order to quantify the number of parasites. Studies have been performed on *Leishmania* parasite loads in mammals [12,13,14] and in sand fly vectors by means of quantitative (q)PCR, as in *Phlebotomus duboseqi* [15], in *Lutzomyia longipalpis* [16,17], and in *Phlebotomus perniciosus* [18]. The phylogenesis and population structure of *Leishmania* strains has been studied by several techniques, such as PCR restriction fragment length polymorphism (PCR-RFLP), sequencing of antigen coding (i.e., *gp63*, hsp70, *cpb*) or noncoding genes (i.e., internal transcribed spacer-1, repetitive DNA sequences), multilocus typing such as multilocus sequence typing (MLST), and multilocus microsatellite typing (MLMT) [19]. 

This study’s main objective was to characterize, by isolation, detection, typing and quantification, the *Leishmania* parasites in sand flies sampled in a site, in the area historically subjected to VL in the Bologna province (Figure 1).

## 2. Materials and Methods 

### 2.1. Sand Fly Sampling and Identification

Sand flies were collected utilizing a carbon dioxide baited trap that operated overnight in a single site in the Valsamoggia municipality, Bologna province (latitude 44.480322°, longitude 11.084237°) (Figure 1). The sampling was started according to the appearance of the first *Leishmania*-positive pool, as detected in the previous surveillance seasons [20]. Collected samples were taken to the laboratory and sorted by the day of sampling. Only a sub-sample of collected insects were tested; after sex sorting, part of the females was grouped into pools (maximum of 100 specimens per pool) and submitted to biomolecular analysis, while other specimens were processed individually (morphologically identified or dissected or utilized for *Leishmania* quantifications). Dissections were done on the day of sampling. Sorted sand flies waiting for analysis and the remaining specimens were stored at –80 °C (Table 1). For each sampling date, some of the males were clarified overnight by chlorolactophenol, mounted with Hoyer’s medium on glass slides and identified under an optical microscope according to morphological characters, reported in the appropriate keys [21,22]. Since morphological identification needs to be preceded by a clarification procedure, the specimens used for this purpose could not be tested by other methods. Only for dissected specimens, morphological identification was performed without the clarification process. Biomolecular identification of specimens subjected to biomolecular analysis or dissection was done by DNA barcoding of the mitochondrial cytochrome c oxidase gene subunit 1 (COI) [23]. The amplicons of about 650 bp were purified with the Agencourt^®^ AMPure® XP PCR Purification Kit (Beckman Coulter Inc., Indianapolis, USA) and sequenced in both directions by CEQ 8000 automated DNA sequencer using the GenomeLab DTCS Quick Start Kit (Beckman Coulter Inc., Indianapolis, USA). The sequence was submitted to Gen Bank (A.N.: MG948469).

### 2.2. Leishmania Detection and Determination of Parasite Load by qPCR

**Figure 2:**
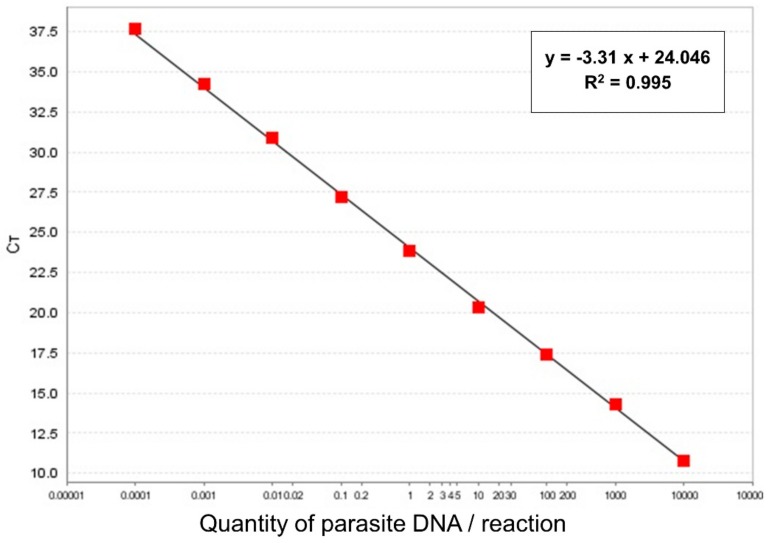
A standard curve was created using 10-fold dilutions of *L. infantum* promastigote DNA. Mean Ct values from five replicates are reported on the Y axis, while the quantity of parasite DNA (ranging from 10,000 to 0.0001 promastigotes per reaction) is reported on the X-axis.

Each specimen or pool was placed in a 1.5 mL tube (Eppendorf), suspended in 250–400 µL of PBS and ground by pellet-pestle (Eppendorf). Starting from 200 µL of a homogenized sample, DNA was extracted using Qiagen DNeasy^®^ Blood & Tissue Kit (Qiagen, Hilden, Germany) according to the manufacturer’s instructions; DNA was eluted in a final volume of 60 μL elution buffer. Obtained extracts were tested for the presence of Leishmania by the Taqman® MGB Real-Time PCR, targeting *Leishmania* minicircle kinetoplast DNA (kDNA), with some modifications [14]. A typical 25 µL reaction mixture contained 5.5 µL of 5× QuantiFast Pathogen Master Mix (Qiagen, Hilden, Germany). The thermal profile was 95 °C for 5 min, followed by 45 cycles of 95 °C for 15 s and 60 °C for 30 s. A standard curve was created to estimate the number of parasites contained in each *Leishmania* positive sand fly. In brief, genomic DNA from a fresh culture of *L. infantum* (MHOM/TN/80/IPT1), with 1 × 10^9^ promastigotes/mL, was extracted using the Qiagen DNeasy® Blood & Tissue Kit (Qiagen, Hilden, Germany), followed by a 1/5 dilution of stock DNA. Successively, amplification of serial dilutions of DNA, ranging from 100,000 to 0.0001 parasites per reaction (in 5 µL), was carried out by five replicates per dilution, allowing the construction of a standard curve. Using StepOne Software v2.3, the standard curve showed a slope of −3.31 and an R^2^ of 0.995 (Figure 2).

**Figure 3:**
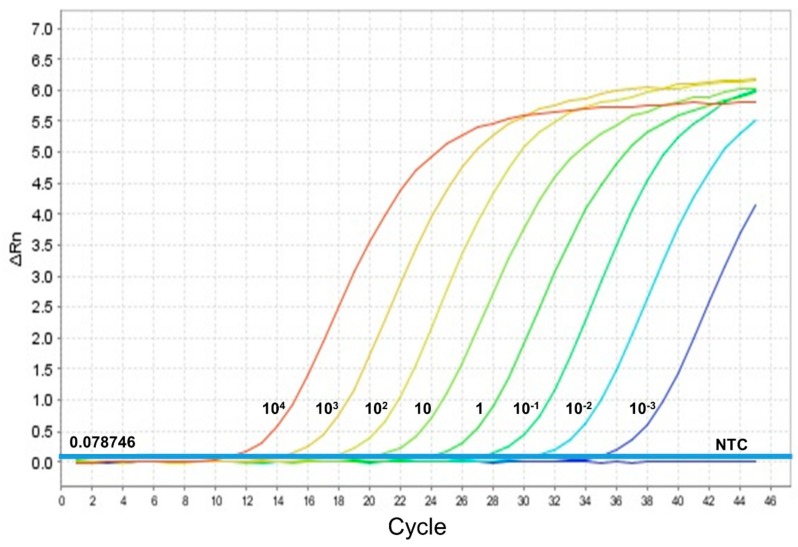
Amplification curves of each promastigote DNA dilution (promastigotes/reaction). One promastigote/mL corresponds to 0.001 promastigotes/reaction, NTC (no template control) and the threshold 0.078749 are shown.

The limit of detection (LOD) was 0.001 parasites per reaction, which corresponds to 1 parasite per mL from the original parasite suspension, at a mean Ct value of 34.7 (Figure 3).

On the base of the haploid genome size of *L. infantum*, one parasite yields approximately 80.2 fg of DNA [24]. Based on this, our quantitative kDNA Real-Time PCR (qPCR) assay achieved 0.08 fg of parasite DNA per reaction (0.001 parasite/reaction × 80.2 fg).

To quantify the parasitic DNA load present in each *Leishmania* positive sand fly, a standard curve of *Leishmania* DNA serial dilutions, ranging from 1,000 to 0.001 parasites per reaction, was carried out by triplicates in each experiment and the parasite load was estimated as the quantified number of parasites (QPN). We considered a single sand fly to be infected if ten or more parasites per specimen were estimated.

### 2.3. Leishmania Isolation and Typing

A part of the collected females, randomly selected, were stunned and dissected. The number of dissected females relied on the available staff working on the procedure. Guts of dissected insects were examined by optical microscopy for detection of motile promastigotes. When protozoa were detected by microscopy, isolation was performed by employing Evans modified Tobie’s medium containing 15% defibrinated rabbit blood. Species identification of the isolated protozoa was performed by amplification and sequencing of the ribosomal DNA Internal Transcribed Spacer-1 (ITS-1) region of 320bp, using the primer pair LITSR/ L5.8S [25], and following the previously described protocols [20], (A.N.: MG969403). In addition, isolated protozoa were investigated by MLMT, employing the following 15 markers: Li41-56, Li46-67, Li21-34, Li22-35, Li23-41, Lm2TG, Lm4TA, Li71-5/2, LIST7039, Li71-33, Li71-7, CS20, Li45-24, TubCA, and LIST7031, as previously reported [26].

Relationships among strains were evaluated by a tree constructed using the Sainudiin model [27] available in the application BEASTvntr package, implemented in the BEAST2 software [28]. The diploid data were entered as two distinct partitions, with a linked tree and a strict clock. A chain length of ten million steps was set.

The *Leishmania* DNA that was detected by kDNA PCR in single sand flies was amplified for a 250 amplicon of a nuclear repetitive region on chromosome 31 (rnr_chr.31), as reported by Piarroux et al. 1995 [29] and sequenced as described above (A.N.: MK765051–MK765060). The sequences obtained were aligned with selected sequences available in GenBank. The phylogenetic tree of the *Leishmania* rnr_chr.31 sequences were inferred using a Bayesian approach with the software BEAST2 for 20 million iterations, and using the substitution model JC69, the strict clock, and a coalescent constant population. All sequences were stored in GenBank and trees were visualized with FIGTREE 1.4.

### 2.4. Captivity of Field Collected Sand Flies

Field collected sand flies were transferred alive into glass jars, kept in a climatic chamber (at 25 °C, 75% HR, 12 h light) and fed daily with sucrose solution. After one week, the jars were washed with sterile water, which was transferred into a 50 mL tube (Falcon) and centrifuged. Supernatants were discharged and pellets were resuspended in about 1 mL of sterile water, transferred into a 1.5 mL tube and tested by kDNA PCR for *Leishmania* spp. as above. Captive sand flies were pooled according to sex with a maximum of 25 specimens per pool and tested by kDNA PCR as above. These insects were collected according to the described protocol, in the same site, in season following the other samplings.

## 3. Results

### 3.1. Leishmania Isolation from Sand Fly and Species Definition

A total of 6114 sand flies were collected in the period from July 27th to September 14th, 2016. Of these, 428 randomly chosen males were morphologically identified as *P. perfiliewi* (96.5%) and *P. perniciosus* (3.5%) (Table 1).

Among the 30 female pools examined, 20 resulted positive for *Leishmania* DNA, with evidence of an increasing rate of positive pools during the sampling period, especially in August; all sand fly pools were *Leishmania*-positive in August (Table 1).

Part of the females collected on July 27 (128) and on August 31 (94) were stunned and dissected. Guts of dissected insects were examined by microscopy, and motile promastigotes were detected in a female from the latter sampling. This dissected female was morphologically identified as *P. perfiliewi*, as confirmed by COI sequencing (A.N.: MG948469). The protozoa detected by microscopy were placed in a culture and successfully isolated. Isolated protozoa were identified as *L. infantum* (hereinafter, Lein-pw) by the ITS-1 sequencing (A.N.: MG969403).

### 3.2. Lein-pw Molecular Typing

**Figure 4:**
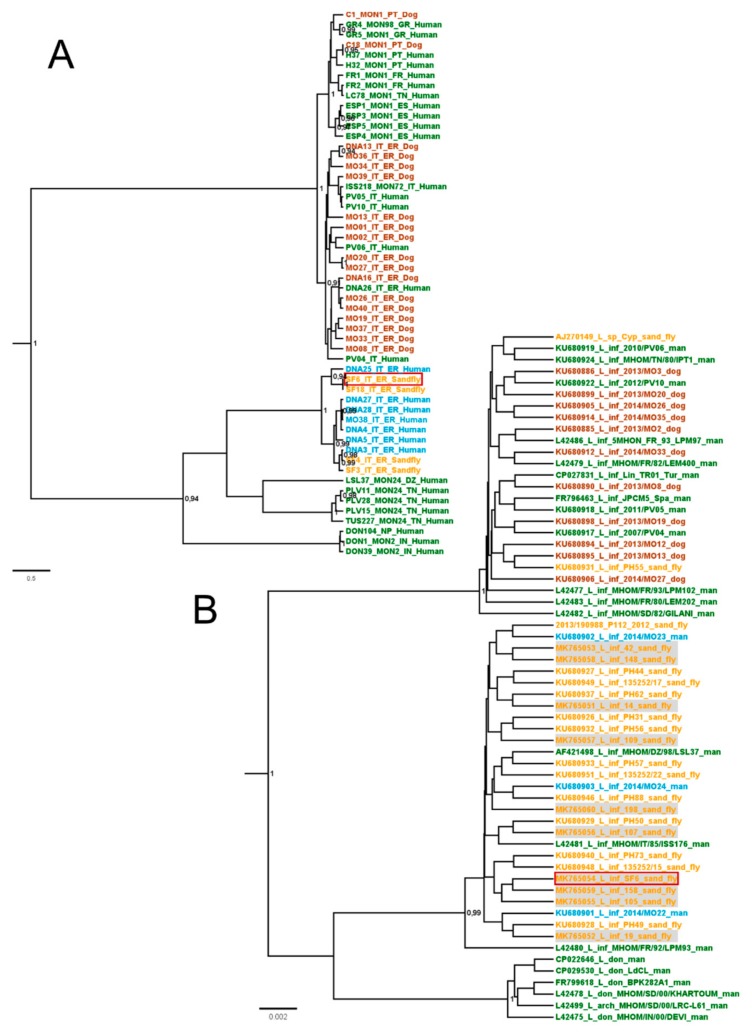
Bayesian trees obtained by multilocus microsatellite typing (MLMT) (**A**) and by analysis of the repetitive nuclear region on chromosome 31 (**B**) of strains of *Leishmania* from the monitored area and other selected strains. Posterior probabilities over 0.9 showed near the respective node; human strains from the Emilia-Romagna region in azure, human strains obtained from outside the Emilia-Romagna region in green; canine strains in brown, sand fly strains in yellow; sequences obtained in this study are marked in grey, Lein-pw is surrounded by a red square.

Lein-pw DNA was examined by MLMT obtaining the following allelic results (reported in brackets): Li41-56 (9), Li46-67 (7), Li21-34 (8), Li22-35 (9), Li23-41 (12), Lm2TG (10), Lm4TA (9), Li71-5/2 (9), LIST7039 (17), Li71-33 (11), Li71-7 (11), CS20 (15), Li45-24 (7), TubCA (10), and LIST7031 (8). This profile was compared to other strains of different origin (Figure 4); the analysis showed that Lein-pw was closely related to *Leishmania* parasites from VL cases in the Emilia-Romagna region, while it was more distantly related to parasites isolated from dogs in the same region in line with previous findings [10,26] (Figure 4). Moreover, Lein-pw was related to the MON-24 strain from northern Africa (Figure 4A). These findings were confirmed by the analysis of the nuclear repetitive region on chromosome 31 of the Lein-pw strain (A.N.: MK765054) (Figure 4B). Interestingly, the Lein-pw sequence was also observed in ten infected sand fly females, collected in the field and individually tested in this study by molecular methods (A.N.: MK765051-MK765053, MK765055-MK765060).

### 3.3. Infection Rate in Sand Flies and Parasite Load by qPCR

In order to more precisely define the sand fly infectious rate and the parasite load, 380 sand flies (253 females and 127 males) randomly chosen from different sampling days were individually tested and the number of parasites per specimen was estimated by using the obtained kDNA standard curve (Table 2). Quantification of parasites showed that not all specimens bore a whole parasite (Table 3).

A total of 68 females (26.9%) tested positive to *Leishmania* DNA, but only 14 bore more than one parasite (from 7 to 210,000 parasites estimated per sand fly) (Table 2 and Table 3).

Surprisingly, 14 males also tested positive for *Leishmania* DNA, but none harbored a whole parasite (Table 2 and Table 3). The presence of ten estimated parasites was arbitrarily set to consider a sand fly specimen as infected. Accordingly, the infectious rate in females ranged from 6% to 10%, with evidence of an increasing trend during the season (Table 2).

Molecular identification by COI sequencing was successful in 68 *Leishmania* positive sand flies (56 females and 12 males), of which 67 were *P. perfiliewi* and one was a *P. perniciosus* (a low-burden positive female).

Additional sampling was performed the following season (summer 2017) in order to evaluate the possible presence of kDNA in sand fly faeces and excreta. 408 alive sand flies were collected and kept in captivity in 4 glass jars. The waste matter laid by sand flies in jars was recovered by washing; the waste matter recovered from 3 out of 4 jars tested positive for *Leishmania* DNA (Table 4), confirming the elimination of kDNA by infected insects. Captive insects were also tested in pools and *Leishmania* DNA was quantified by qPCR, estimating more than 10 *Leishmania* parasites in 3 out of 6 pools of females (Table 4).

## 4. Discussion

Our data confirm that *P. perfiliewi* is the most abundant sand fly species in the Bologna province (Emilia-Romagna region, northeastern Italy), as previously reported [4,10,11]. In the monitored area, *P. perfiliewi* was largely more abundant than *P. perniciosus*, the latter being considered the main vector of leishmaniasis in Italy [3]. The vector role of *P. perfiliewi* in the surveyed area was proved by the isolation of a *L. infantum* strain from a *P. perfiliewi* female and by the detection of *P. perfiliewi* females carrying a high-burden of *Leishmania* parasites. Consistently, the vector role of *P. perfiliewi* in the Emilia-Romagna was not excluded in studies conducted in the same area in the 70s [10,11].

Interestingly, the isolated parasitic strain (Lein-pw) was closely related to *Leishmania* parasites from VL human cases in the Emilia-Romagna region, but differed from strains isolated from dogs in the same area [10,26] (Figure 4). The lack of detection of this *L. infantum* strain in dogs raises doubts of the primary role of the canine reservoir in the local eco-epidemiological situation. In line with this hypothesis, a low *Leishmania*-seroprevalence was recorded in dogs in the Valsamoggia municipality, where the site sampled in this study was located. In 2016, less than 1% of tested dogs were seropositive, with 7 positive samples out of 1325 canine sera tested by indirect immunofluorescence assay (Dr. Michele Ottavio Sabatino, personal communication). Dermotropic *L. infantum* strains closely related to Lein-pw (Figure 4) were previously isolated in northern Africa, specifically in Algeria (strains marked with DZ in Figure 4) and Tunisia (strains marked with TN in Figure 4). In line with our findings, the main sand fly present in the abovementioned studies from northern Africa was *P. perfiliewi*, and no obvious animal reservoir was identified to date in these areas [30,31,32,33]. Although the dog is considered the main reservoir of *L. infantum* in the Mediterranean basin, other animals such as red foxes, hares, rats, martens, and badgers were found infected in nature, which leads to hypothesize a reservoir role of wildlife for this parasite [34], particularly in anomalous ecological situations. This appeared to be the case in the recent outbreak of human leishmaniasis in Spain, which was caused by overabundant populations of sand flies and hares (acting as a reservoir in this context), in close contact with humans [35]. We suggest that the incrimination of *P. perfiliewi* as a vector can prelude to other peculiarities in the local cycle of *L. infantum*, as the involvement of reservoirs other than dogs. In light of the foregoing, studies targeting potential wild animal reservoirs, such as hares, roe deer, mice, rats, hedgehogs, and foxes, are ongoing to clarify the epidemiological cycle sustaining the endemic presence of the parasite in the study area. In addition, evaluation of blood meal of engorged females is ongoing to identify the host preference of sand flies in the study area.

Studies to detect the prevalence of *Leishmania* in their vectors are of crucial importance and several PCR-based tools have been developed [36]. A qPCR targeting kDNA [12] was employed to estimate the parasite load in sand fly vectors. In line with previous results, the use of this PCR obtained a LOD value of 0.001 parasites per reaction [16,18]. The high sensitivity of the kDNA PCR is due to the extensive number of copies of the target minicircle kDNA molecules per parasite (approximately 10,000 copies) [12,13,16] and allows for the detection of a quantity of kDNA targets that are below one parasite per sand fly.

The prevalence of *Leishmania* infection in *P. perfiliewi* sand flies varied from 6% to 10% with evidence of an increasing trend during the sampled seasonal period. A similar prevalence was reported in sand flies collected in a focus of VL outbreak in Spain, even if supported by *P. perniciosus* as a vector [36,37]. Such a high infection rate raises questions on the transmission mechanisms supporting this phenomenon in natural conditions. To elucidate the seasonal dynamic of the infection rate, regular collection and analysis of sand flies during the whole favorable period could be useful.

The presence of sand flies that are estimated to harbor less than one parasite raises the issue of the correct estimation of infection rates in field-collected sand flies when using PCR methods. The lower than one parasite burden may result from the environmental presence of *Leishmania* genetic material, due to the physiological elimination of the parasites by infected sand flies through urine, feces or other physiological processes. Failure to escape the peritrophic membrane by parasites may result in their expulsion through defecation, along with the remnants of the blood meal [38]. Expulsion of *Leishmania* promastigotes was reported in artificially-infected sand flies during the pre-diuresis phase, slightly before the blood meal [39]. We suggest that this phenomenon can also occur in field conditions, not necessarily linked to blood meal elimination or the pre-diuresis phase, since the insects caught in the entomological traps (baited by carbon dioxide) were mainly host seeking-females. Such a phenomenon deserves further investigation since it could be relevant when assessing the infection rate of sand flies tested in pools as a potential cause of contamination that could occur in the environment, but also in the trap collection bag.

## 5. Conclusions

Our data suggest the existence of a *L. infantum* cycle involving *P. perfiliewi* as the main vector in the Bologna province, northeastern Italy. This parasitic cycle is characterized by features that seem to differ from the canonical parasitic cycle described in the rest of Italy, which includes dogs as the crucial reservoir hosts. Studies are ongoing to better elucidate the local parasitic cycle and potential reservoir hosts.

## Figures and Tables

**Table 1:** Sampled sand flies in the Valsamoggia municipality (Bologna province, north-eastern Italy), July-September 2016.

Date	N	Tested in Pool	Individually-Tested	pw/pe	Leish-Pos Pools/Total Pools
27-07-16	2728	1000	209	95/5	1/10
11-08-16	431	300	57	16/2	3/3
18-08-16	821	500	80	101/4	5/5
31-08-16	1084	500	176	100/3	5/5
14-09-16	1050	700	80	101/1	6/7
	6114	4030	602	413 (96.5%)/15 (3.5%)	20/30

N, number of collected specimens; Tested in pool, specimens employed for diagnostic PCR; Individually-Tested, specimens employed for morphological identification, or dissection, or *Leishmania* quantifications; pw/pe, *P. perfiliewi*/*P. perniciosus*; Leish-pos pools/total pools, female pools resulted *Leishmania* positive on tested pools.

**Table 2:** Individually-tested sand flies with reference to dates of sampling, number of tested specimens, and amount of parasites inside each *Leishmania*-positive insect.

Date		Female			Male	
	N	<10 (%)	≥10 (%)	N	<10 (%)	≥10
27-07-16	51	6 (11.8)	-	30	-	-
11-08-16	52	5 (9.6)	-	5	-	-
18-08-16	50	20 (40.0)	3 (6.0)	30	3 (10.0)	-
31-08-16	50	19 (38.0)	4 (8.0)	32	11 (34.4)	-
14-09-16	50	6 (12.0)	5 (10.0)	30	-	-
-	253	56 (22.1)	12 (4.7)	127	14 (11.0)	-

N, number of tested specimens; <10, *Leishmania* positive specimens with less than 10 parasites in the sand fly; ≥10, *Leishmania* positive specimens with more than 10 parasites in the sand fly; - no *Leishmania*- positive sand flies.

**Table 3:** Estimation of the number of parasites in *Leishmania*-positive individually-tested sand flies with reference to sex and date of sampling.

Date	Sex	<0.01	0.01–0.1	0.1–1	1–10	10–10^2^	10^2^–10^3^	10^3^–10^4^	10^4^–10^5^	>10^5^
27-07-16	F	5	1							
11-08-16	F		3		2					
18-08-16	F	18	1	1				2	1	
18-08-16	M		3							
31-08-16	F	5	10	4		2	1		1	
31-08-16	M	3	5	3						
14-09-16	F	3	2	1					2	3
Total		34	25	9	2	2	1	2	4	3

F, female; M, male.

**Table 4:** Test on captive sand flies and laid waste matter.

		Faeces and Excreta	Females		Males	
Jar	Sampling Date	QPN	N/N Pools	Positive Pools (QPN)	N/N Pools	Positive Pools (QPN)
1	17-08-17	0.3	142/6	2 (945, 6000)	62/3	1 (0.5)
2	29-08-17	7	71/3	2 (2, 2621)	21/1	-
3	29-08-17	0.1	58/3	1 (0.5)	23/1	-
4	13-09-17	-	5/1	1 (0.05)	26/1	1 (0.05)

QPN, number of parasites estimated by qPCR; N, number of sand flies; N pools, numbers of pools.
